# Coronary Microcirculatory Function Indicated by Coronary Angiography-Derived Index of Microvascular Resistance in Patients Undergoing Rotational Atherectomy

**DOI:** 10.31083/j.rcm2310330

**Published:** 2022-09-28

**Authors:** Hui Li, Xi Peng, Le Li, Yun-Di Feng, Guo-Dong Tang, Ying Zhao, Guo-Jian Yang, Nai-Xin Zheng, Fu-Cheng Sun, Hu Ai, Hui-Ping Zhang

**Affiliations:** ^1^Department of Cardiology, Beijing Hospital, National Center of Gerontology; Institute of Geriatric Medicine, Chinese Academy of Medical Sciences, 100730 Beijing, China; ^2^Department of Cardiology, Fuwai Hospital, National Center for Cardiovascular Diseases, Peking Union Medical College and Chinese Academy of Medical Sciences, 100037 Beijing, China; ^3^PKU-HKUST Shenzhen-Hong Kong Institution, 518063 Shenzhen, Guangdong, China

**Keywords:** coronary artery disease, percutaneous coronary intervention, rotational atherectomy, coronary microcirculation, index of microvascular resistance

## Abstract

**Background:**

There are scarce published data reporting the effect of rotational atherectomy (RA) on coronary microcirculation function.

**Objectives:**

We aimed to evaluate coronary microcirculation function indicated by the coronary angiography-derived index of microvascular resistance (caIMR) in patients undergoing RA.

**Methods:**

RA procedures between January 2013 and December 2021 were retrospectively analyzed. We investigated coronary microcirculation function indicated by caIMR as well as peri-procedural adverse events among the study population. All caIMR measurements were performed using a FlashAngio system. The primary outcome was a composite of post-RA thrombolysis in myocardial infarction (TIMI) flow grade <3 in the target vessel, myocardial injury, procedure-related myocardial infarction, and cardiac death during hospitalization.

**Results:**

A total of 155 RA procedures were analyzed. The post-RA caIMRs were significantly higher than pre-RA caIMRs in the target vessels (16.0 ± 7.0 vs. 14.5 ± 7.5, *p *= 0.029). Patients with post-RA caIMR ≥25 accounted for nearly 12% of those with pre-RA caIMR <25. Patients with post-RA thrombolysis in myocardial infarction (TIMI) flow grade <3 had a significantly higher pre-RA caIMR (23.5 ± 10.2 vs. 13.7 ± 6.6, *p *= 0.005), and the proportion of patients with pre-RA caIMR ≥25 in the group with TIMI flow grade <3 was greater (61.5% vs. 38.5%, *p <* 0.001) than that in the group with TIMI flow grade of 3. Maximum RA time of each pass (odds ratio: 1.127, 95% confidence interval: 1.025–1.239, *p *= 0.014) and pre-RA caIMR ≥25 (odds ratio: 3.254, 95% confidence interval: 1.054–10.048, *p *= 0.040) were identified to be the independent predictors of the primary outcome for patients who underwent RA.

**Conclusions:**

There were significant changes in the coronary microcirculation function of the target vessels after receiving RA as indicated by increased post-RA caIMR compared to pre-RA caIMR. Patients with baseline coronary microcirculatory dysfunction were more likely to have post-RA TIMI flow grade <3, whereas those with pre-RA caIMR ≥25 experienced worse outcomes.

## 1. Introduction

The effect of percutaneous coronary intervention (PCI) on coronary microvascular function and the prognostic implication of pre and post-procedural index of microvascular resistance (IMR) has been shown in previous studies [1, 2, 3]. Coronary rotational atherectomy (RA) is an efficient way to facilitate balloon or stent delivery and optimize stent expansion by physical removal of hard plaque via lumen enlargement [4, 5]. Current PCI guidelines state that RA is a reasonable approach for the treatment of heavily calcified plaques that cannot be crossed by a balloon catheter or adequately dilated before stent implantation [6]. However, the RA procedure has previously been reported to be associated with microvascular disorder resulting from microcirculatory obstruction [5].

The pressure-temperature wire-derived coronary flow reserve (CFR) and IMR have constituted the reference standard to assess the status of coronary microcirculation thus far [7, 8]. Prior studies have indicated that there are major limitations to the pressure wire-derived CFR calculation; the maximal hyperemic coronary blood flow is strongly pressure-dependent, and the pressure wire-derived method appears to systematically underestimate the CFR values [9]. However, the pressure-temperature wire-derived IMR shows good specificity and reproducibility compared with CFR [10, 11], whereas the invasive measurement increases additional intracoronary performance and prolongs the operation time. Thus, to a certain degree, the invasive measurement raises unpredictable procedural risks, especially when faced with treating complicated lesions or in urgent situations. Alternatively, multiple pressure-wire-free tools, such as angiography-derived index of microcirculatory resistance, to assess coronary microvascular dysfunction have been developed [12]. The pressure-wire-free method was revealed to be well correlated with wire-derived IMR for estimation of microcirculatory function [13]. A novel coronary angiography-derived index of microvascular resistance (caIMR) shows good agreement with pressure-temperature wire-based IMR and has similar accuracy; thus, it has been proposed as a well-adopted non-invasive physiological assessment of coronary microcirculation function [14, 15, 16]. The effect of RA on coronary microcirculation function remains unclear, and this study aimed to investigate coronary microcirculatory function indicated by caIMR in patients undergoing RA.

## 2. Materials and Methods

### 2.1 Study Population

Between January 2013 and December 2021, consecutive RA procedures in patients with severe coronary artery calcification lesions and significant stenosis (stenosis ≥75% of the vessel diameter) from a dedicated RA database were retrospectively analyzed. Severe coronary artery calcification was defined visually with fluoroscopy as the presence of radio-opacities within the vessel wall without cardiac motion before contrast injection or determined by the presence of ≥270° of high-intensity echoes with acoustic shadowing at one cross-section on intravascular ultrasound (IVUS) [17, 18]. Patients who underwent chronic total occlusion PCI and patients with acute coronary syndrome (ACS) who underwent primary PCI or urgent invasive PCI were excluded. Considering the requirements of high-quality coronary angiographic images, RA procedures containing unclear contrast opacification, marked vascular overlap or distortion of the targeted vessel, or poor-quality angiographic images which were difficult to analyze were excluded from the study. All patients were treated with 100–300 mg aspirin and a loading dose of 300 mg of clopidogrel before the procedure. Dual antiplatelet therapy was continued for at least 12 months, followed by mono antiplatelet therapy with aspirin (100 mg/day) or clopidogrel (75 mg/day) indefinitely. The study was approved by the institutional ethics committee, and all patients provided written informed consent to undergo coronary angiography and the intervention procedure. Since caIMR data were collected retrospectively, informed consent for the use of caIMR was waived according to the institutional ethics regulations with regard to the observational nature of this study.

### 2.2 RA Procedure

Coronary RA was performed using a Rotablator Rotational Atherectomy System (Boston Scientific, Marlborough, MA, USA). Standard techniques for PCI were performed by an experienced operator. The most widely adopted institutional protocol for rotablation was used. The preferred burr-to-artery ratio was 0.5, and a smaller (1.25-mm) burr was initially used more often, followed by a larger (1.50-mm, 1.75-mm) burr. Before approaching the target lesion, the burr advanced at a low speed of 60,000 to 70,000 revolutions per minute (rpm). The working rotational speed of the burr ranged from 130,000 to 180,000 rpm. When the target lesion could not be fully dilated, a higher-speed (≥180,000 rpm) atherectomy was performed. Each pass was limited to ≤30 seconds. During the RA procedure, patients received unfractionated heparin with an initial bolus of 80–100 U/kg and additional boluses of 1000 U/h. A Rota-flush solution contains 12,500 units of unfractionated heparin, 5 mg of verapamil, and 5 mg of nitroglycerin in a 1-L bag of saline solution.

The RA was performed when the target lesion was deemed undilatable by a balloon based on angiography and/or IVUS findings indicated the requirement of planned RA. RA procedures performed when it was not possible to fully expand the target lesion were regarded as rescue RA. The use of IVUS for the evaluation of lesion features and stent expansion was left to the discretion of the operator. After RA, patients received pre-dilation with conventional, scoring, or cutting balloons, as determined by the operator. When adequate pre-treatment results were achieved, one or more drug-eluting stents were implanted.

### 2.3 Peri-Procedural Adverse Events

We established a dedicated RA database to record demographic, angiographic, and procedural data, including characteristics of RA and peri-procedural events, as well as hospitalization information. Peri-procedural adverse events (PPAEs) including coronary slow flow or no flow post-RA, coronary dissection, burr entrapment, side branch occlusion, peripheral vascular complications, contrast-induced nephropathy, procedure-related myocardial infarction (MI), and in-hospital death were recorded. Coronary slow flow/no flow refers to instant thrombolysis in myocardial infarction (TIMI) flow grade <3 after the RA procedure without visible thrombosis, dissection, or spasm. Procedure-related MI was defined as elevation of cardiac troponin (cTn) >5 times the upper limit of normal and recurrent symptoms with or without new ST-segment changes. An increase of cTn values in patients with normal baseline values or a rise of cTn values >20% of the baseline were regarded as myocardial injury [19]. The primary outcome was a composite of post-RA TIMI flow grade <3 in the target vessel, myocardial injury, procedure-related MI, and cardiac death during hospitalization.

### 2.4 caIMR Measurement

A three-dimensional mesh of the target artery was reconstructed based on two coronary angiographic projections which were at least 30° apart and had no vessel overlap. In theory, the caIMR (unit: mmHg⋅s/mm) was computed as follows:

(1) $$\mathrm{caIMR}={\mathrm{Pd}}_{\text{hyp }}\,\frac{L}{K\cdot V_{\text{diastole }}}.$$

In the above equation, ${\mathrm{Pd}}_{\text{hyp}}$ is the mean distal coronary pressure at the maximal hyperemia. The hyperemic Pd was calculated via the Navier-Stokes equation. A specially designed computational fluid dynamics model for the steady-state laminar flow has been previously described in detail [20]. This method was used to compute the pressure drop (Δ$P_{\text{hyp}}$) along meshed coronary arteries from the inlet to the distal coronary artery (${\mathrm{Pd}}_{\text{hyp}}$ [unit: mmHg] = ${\mathrm{Pa}}_{\text{hyp}}$–Δ$P_{\text{hyp}}$). ${\mathrm{Pa}}_{\text{hyp}}$ represents the maximal hyperemic mean aortic pressure, which was computed by averaging the pressure waves in three cardiac cycles. ${\mathrm{Pa}}_{\text{hyp}}$ is calculated using a mathematical formula expatiated in previous studies [14, 20]. L is a non-dimensional constant that simulates the length measured from the inlet to the distal artery, and it is generally 75 representing a 75-mm distance downstream from the coronary inlet. $V_{\text{diastole}}$ (unit: mm/s) is the mean blood flow velocity at diastole, and it is indicative of the contrast passing length (mm)/diastolic time interval (s). The contrast passing length can be calculated as the distance moved by the contrast medium in three-dimensional reconstructed coronary arteries during the diastolic period. K is a constant (K = 2.1), and K⋅$V_{\text{diastole}}$ is assumed to be the maximal hyperemic flow velocity [21, 22]. The caIMR computation was performed using a FlashAngio system (Rainmed Ltd, Suzhou, China), and the measurements were performed by blinded operators. In the target vessels, caIMR was calculated at the stage of before PCI and after finalizing PCI. In reference vessels, caIMR was obtained at the stage of before PCI. Considering the potential impact of wedge pressure resulted from collateral flow on caIMR under the circumstance of severe stenosis being present, a corrected caIMR following Yong’s formula was calculated in all patients [23].

### 2.5 Statistical Analysis

Continuous variables are expressed as the mean ± standard deviation or median (interquartile range), as appropriate. Categorical variables are presented as numbers and percentages. The chi-square test or Fisher’s exact test was used for the comparison of categorical variables. The Student’s *t* test or Mann-Whitney rank-sum test was used to test differences among continuous variables based on their distributions. A multivariable analysis using a logistic regression model was conducted to determine predictors of the primary outcome, and the results are expressed as odds ratios (ORs) with 95% confidence intervals (CIs). Variables suggested to be related to the outcome of interest according to clinical consideration and with *p *< 0.05 in the univariate analysis were adopted as candidate predictors for the multivariate analysis. Two-tailed *p *< 0.05 was considered statistically significant for all tests. All statistical analyses were performed using SPSS version 20.0 (IBM Corporation, Armonk, NY, USA).

## 3. Results

### 3.1 Baseline, Angiographic, and Procedural Characteristics

A total of 192 consecutive patients who underwent RA between January 2013 and December 2021 were enrolled in this study. Thiry-seven patients with coronary angiography involving unclear contrast opacification, marked vascular overlap or distortion of the targeted vessel, poor-quality angiographic images, or lack of two images that were ≥30° apart were excluded. In the final analysis, 155 RA procedures were included. Detailed clinical baseline, angiographic and procedural characteristics are shown in Table 1. All patients had a normal TIMI flow grade before the RA procedure. The left anterior descending artery (LAD) accounted for most cases of treated arteries (127, 81.9%). The percentage of post-RA myocardial injury was 23.9% (n = 37). Most PPAEs were minor and without unfavorable prognoses. The common PPAEs were procedure-related MI (17, 11%) and post-RA TIMI flow grade <3 (13, 8.4%). The occurrence of TIMI flow <3 was more often observed in LAD (10/13, 76.9%). Burr entrapment occurred in two (1.3%) patients and was successfully relieved by repeat balloon dilation following removal of the whole RA system. One (0.6%) patient with a left ventricular ejection fraction of 20% died due to refractory heart failure during hospitalization. No cardiac tamponade occurred, and no definite or probable stent thrombosis was recorded in any of the patients.

**Table 1.** **Clinical baseline, angiographic and procedural characteristics for the study population**.

**Variables**		**n = 155**
**Clinical baseline characteristics**		
Age (years)		70.1 ± 9.1
Male gender		94 (60.6)
Hypertension		118 (76.7)
Diabetes mellitus		76 (49.0)
Current smoker		52 (33.5)
Previous MI		22 (14.2)
Prior PCI		72 (46.5)
UAP		87 (56.1)
eGFR (mL·$\min^{–\,1}$·${\mathrm{1.73}}^{–\,1}$)		73.8 ± 23.6
LVEF		61.1 ± 9.2
**Angiographic and procedural characteristics**		
PCI access		
	Transradial	126 (81.3)
	Transfemoral	29 (18.7)
Three-vessel coronary disease		112 (72.2)
Contrast volume (mL)		266.5 ± 86.5
Target vessel		
	LAD	127 (81.9)
	LCX	7 (4.5)
	RCA	21 (13.5)
≥20 mm lesion		128 (82.6)
Bifurcation lesion		84 (54.2)
Planned RA		109 (70.3)
Rescue RA		46 (29.7)
IVUS use		73 (47.1)
Number of rotational times		4 (3, 5)
Maximum RA time of each pass (seconds)		17.0 ± 3.9
Maximum rotational speed (10,000 rpm)		15.8 ± 1.3
Number and size of burrs		
	1	138 (89)
	2	17 (11)
	1.25 mm	103 (59.9)
	1.50 mm	68 (39.5)
	1.75 mm	1 (0.6)
Number of stents		2 (2, 2)
Myocardial injury		37 (23.9)
Peri-procedural adverse events		33 (21.3)
Instant TIMI flow grade <3		13 (8.4)
No flow		1 (0.6)
Procedure related-MI		17 (11)
In-hospital death		1 (0.6)
The primary outcome		61 (39.3)

Values are mean ± standard deviation, median (interquartile range) or n (%). MI, myocardial infarction; PCI, percutaneous coronary intervention; UAP, unstable angina pectoris; eGFR, estimated glomerular filtration rate; LVEF, left ventricular ejection fraction; LAD, left anterior descending artery; LCX, left circumflex; RCA, right coronary artery; RA, rotational atherectomy; IVUS, intravas- cular ultrasound; rpm, revolutions per minute; TIMI, thrombolysis in myocardial infarction. The primary outcome was a composite of TIMI flow <3 post-RA in the target vessel, myocardial injury, procedure related MI, and cardiac death during hospitalization.

### 3.2 caIMR, Myocardial Injury, and Procedure-Related MI

**Fig. 1.**
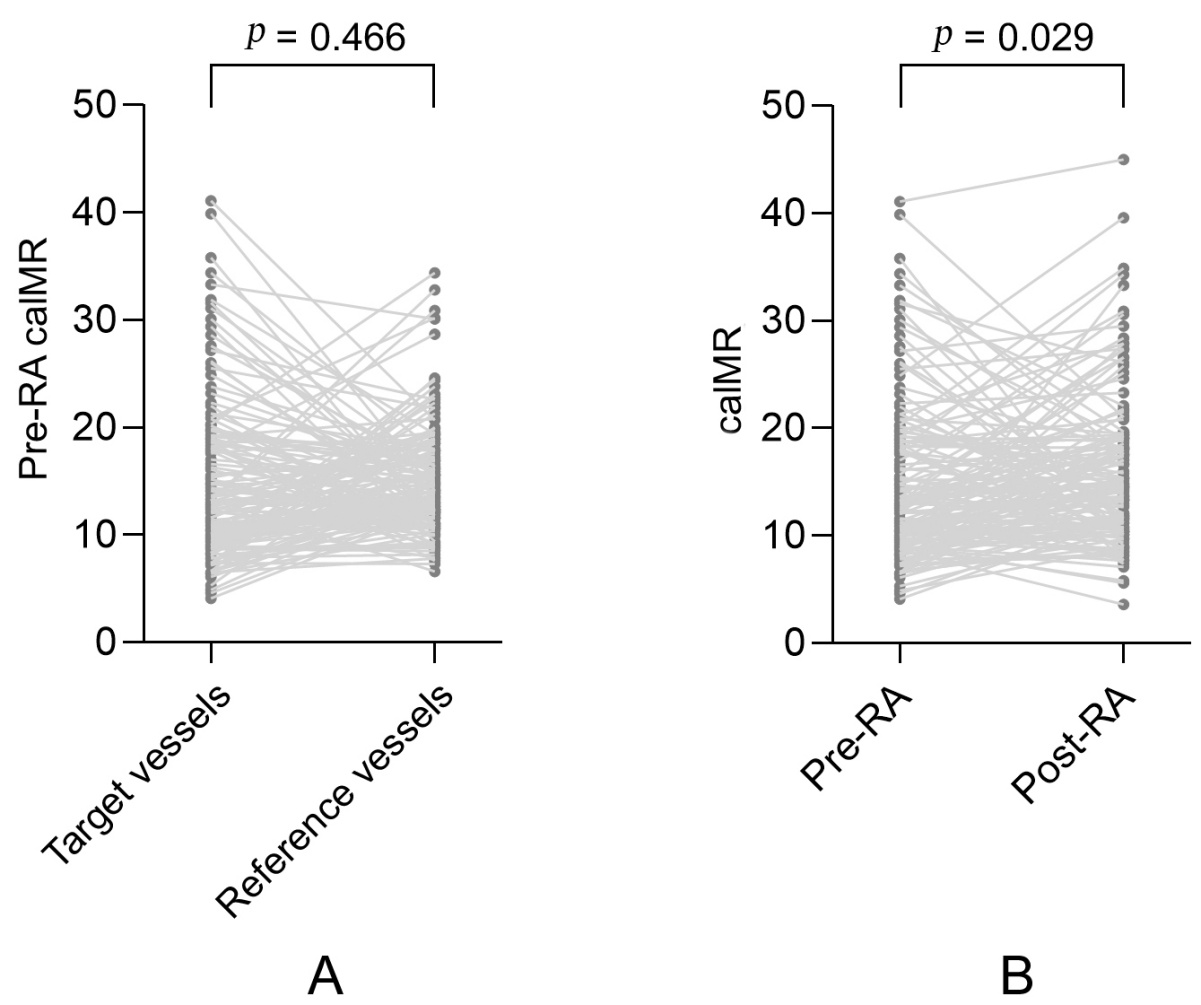
**A paired comparison of caIMR in the target and reference vessels**. (A) A paired comparison of pre-RA caIMR between the target and reference vessels. (B) A paired comparison of pre-RA and post-RA caIMR in the target vessels. MI, myocardial infarction; caIMR, coronary angiography-derived index of microvascular resistance; RA, rotational atherectomy.

**Fig. 2.**
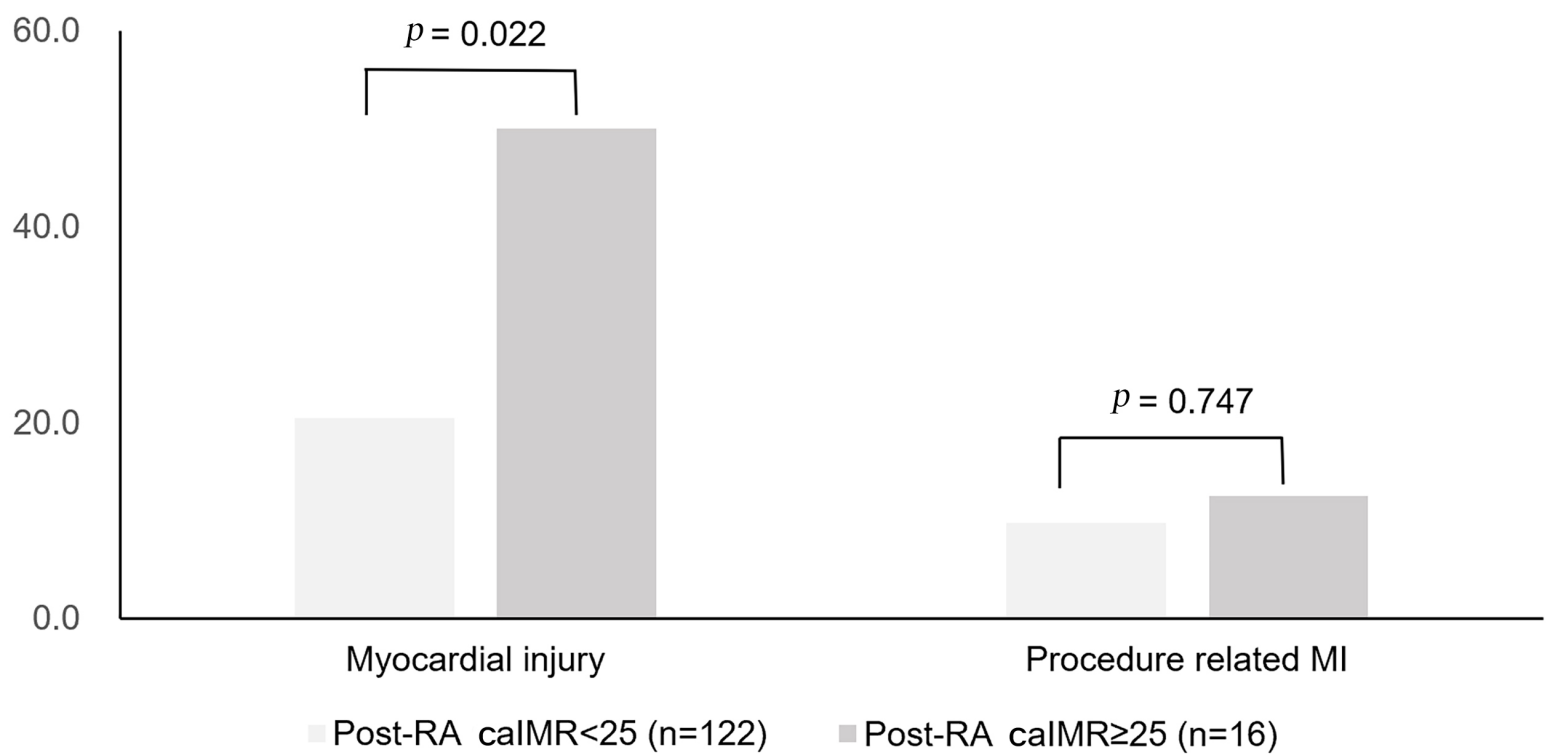
**Occurrences of myocardial injury and procedure-related MI among patients with pre-RA caIMR <25**. MI, myocardial infarction; caIMR, coronary angiography-derived index of microvascular resistance; RA, rotational atherectomy.

There were no significant differences in pre-RA caIMR measurements between the target and reference vessels (15.2 ± 5.2 vs. 14.6 ± 7.5, *p* = 0.466). However, post-RA caIMRs were significantly higher than pre-RA caIMRs in the target vessels (16.0 ± 7.0 vs. 14.5 ± 7.5, *p* = 0.029), as shown in Fig. 1. Patients with post-RA caIMR ≥25 accounted for nearly 12% (n = 16) of those with pre-RA caIMR <25. Among patients with pre-RA caIMR <25, the incidence of myocardial injury was significantly lower in those with post-RA caIMR <25 than that in those with post-RA caIMR ≥25 [25 (20.5%) vs. 8 (50.0%), *p* = 0.022]. Furthermore, the rates of procedure-related MI were comparable between the two groups [12 (9.8%) vs. 2 (12.5%), *p* = 0.747], as shown in Fig. 2.

### 3.3 caIMR, Myocardial Injury, and Procedure-Related MI Stratified by Post-RA TIMI Flow

Patients with post-RA TIMI flow grade <3 had a significantly higher pre-RA caIMR (23.5 ± 10.2 vs. 13.7 ± 6.6, *p* = 0.005), and the proportion of patients with pre-RA caIMR ≥25 in the group with post-RA TIMI flow grade <3 was greater (61.5% vs. 6.3%, *p <* 0.001) than that in the group with post-RA TIMI flow grade of 3. Similarly, patients with post-RA TIMI flow grade <3 had post-RA higher caIMR (25.6 ± 8.0 vs. 15.1 ± 6.2, *p <* 0.001), and the proportion of patients with post-RA caIMR ≥25 in the group with post-RA TIMI flow grade <3 was greater (53.8% vs. 7.0%, *p <* 0.001). There was no significant difference between the group with post-RA TIMI flow grade <3 and that with TIMI flow grade of 3 concerning the rate of myocardial injury (38.5% vs. 22.5%, *p* = 0.342). More patients had procedure-related MI in the group with post-RA TIMI flow grade <3 than those in the group with TIMI flow grade of 3 (30.8% vs. 9.2%, *p* = 0.040), as summarized in Table 2.

**Table 2.** **caIMR, myocardial injury and procedure-related MI according to post-RA TIMI flow**.

Variables		post-RA TIMI flow grade <3	post-RA TIMI flow grade 3	*p* value
		(n = 13)	(n = 142)	
pre-RA caIMR		23.5 ± 10.2	13.7 ± 6.6	0.005
	≥25	8 (61.5)	9 (6.3)	<0.001
	<25	5 (38.5)	133 (93.7)	
post-RA caIMR		25.6 ± 8.0	15.1 ± 6.2	<0.001
	≥25	7 (53.8)	10 (7.0)	<0.001
	<25	6 (46.2)	132 (93.0)	
Myocardial injury		5 (38.5)	32 (22.5)	0.342
Procedure-related MI		4 (30.8)	13 (9.2)	0.040

Values are mean ± standard deviation or n (%). caIMR, coronary angiography-derived index of microvascular resistance; MI, myocardial infarction; TIMI, thrombolysis in myocardial infarction; RA, rotational atherectomy.

### 3.4 Predictors of Primary Outcome in Patients who Underwent RA

Candidate predictors in the univariate analysis included age, hypertension, diabetes mellitus, previous MI, estimated glomerular filtration rate, number of diseased vessels, lesions ≥20 mm, bifurcation lesion, RA strategy (planned or rescue RA), number of rotational times, maximum RA time of each pass, maximum rotational speed, pre-RA caIMR, post-RA caIMR, and percentage of pre-RA caIMR ≥25 and post-RA caIMR ≥25 in the treated vessels. The variables entered into the logistic regression model were maximum RA time of each pass and percentage of pre-RA caIMR ≥25 and post-RA caIMR ≥25. Table 3 shows multivariate predictors of the primary outcome in patients who underwent RA. The multivariable analysis revealed that the independent predictors of the primary outcome were maximum RA time of each pass (OR: 1.127, 95% CI: 1.025–1.239, *p* = 0.014) and caIMR pre-RA ≥25 (OR: 3.254, 95% CI: 1.054–10.048, *p* = 0.040) for patients who underwent RA.

**Table 3.** **Predictors of the primary outcome in patients who underwent RA**.

Variable	Univariate OR (95% CI)	*p* value	Adjusted OR (95% CI)	*p* value
Age (years)	1.034 (0.997–1.073)	0.075		
Hypertension	1.420 (0.649–3.110)	0.380		
Diabetes mellitus	0.989 (0.518–1.886)	0.973		
Previous MI	1.028 (0.991–1.066)	0.136		
eGFR (mL·$\min^{–\,1}$·${\mathrm{1.73}}^{–\,1}$)	0.990 (0.976–1.004)	0.165		
number of diseased vessels	1.017 (0.985–1.049)	0.305		
lesions ≥20 mm	1.368 (0.571–3.281)	0.482		
bifurcation lesion	0.994 (0.520–1.897)	0.985		
RA strategy (planned or rescue RA)	1.449 (0.720–2.914)	0.298		
number of rotational times	1.127 (0.999–1.271)	0.053		
maximum RA time of each pass (seconds)	1.121 (1.021–1.230)	0.016	1.127 (1.025–1.239)	0.014
maximum rotational speed	1.000 (1.001–1.100)	0.705		
pre-RA caIMR	1.027 (0.984–1.073)	0.219		
pre-RA caIMR ≥25	3.227 (1.125–9.253)	0.029	3.254 (1.054–10.048)	0.040
post-RA caIMR	1.018 (0.973–1.066)	0.441		
post-RA caIMR ≥25	3.592 (1.269–10.166)	0.016	2.834 (0.958–8.386)	0.060

RA, rotational atherectomy; OR, odds ratio; CI, confidence interval; MI, myocardial infarction; eGFR, estimated glomerular filtration rate; caIMR, coronary angiography-derived index of microvascular resistance. The primary outcome was a composite of TIMI flow <3 post-RA in the target vessel, myocardial injury, procedure-related MI, and cardiac death during hospitalization.

## 4. Discussion

The aim of this study was to evaluate coronary microcirculation function indicated by caIMR in patients undergoing RA. Our main findings are as follows: (1) Post-RA caIMR, which indicates coronary microcirculation function, was greater than pre-RA caIMR in the treated vessels. Patients with ≥25 post-RA caIMR accounted for nearly 12% of those with pre-RA caIMR <25; (2) among patients without increased pre-RA caIMR, those with post-RA caIMR ≥25 were associated with a significantly increased incidence of myocardial injury compared to those with post-RA caIMR <25; (3) patients with post-RA TIMI flow grade <3 showed significant differences in both pre- and post-RA caIMR compared with those of patients with normal TIMI flow; (4) among patients who underwent RA, those receiving longer RA time of each pass and with pre-RA caIMR ≥25 had worse outcomes.

It has been demonstrated that RA facilitates procedural success in treating calcified plaques, especially in complex ostial lesions and bifurcation lesions, which feature bulky plaque and unfavorable geometry for stent deployment [24, 25]. While there have always been concerns regarding microcirculatory dysfunction associated with RA, analyzing the CFR and coronary microvascular resistance using intracoronary Doppler guidewire has been considered to be the most reliable method for coronary microcirculation assessment [26, 27]. However, intracoronary Doppler guidewire is unavailable in current practice. The pressure-temperature wire-derived CFR is associated with variations in measurement and has unsatisfied reproducibility [9, 28]. The pressure-temperature wire-derived measurements of coronary microcirculation, indicated by IMR, seem impracticable with regard to real-world applicability, particularly when applied in urgent situations or complex PCI. Previous studies have demonstrated that caIMR is a feasible alternative for the evaluation of coronary microcirculatory function [14, 15, 16].

In the present study, post-RA caIMRs were significantly higher than pre-RA caIMRs in the treated vessels. Nearly 1/8th of patients without demonstrated microcirculatory dysfunction indicated by pre-RA caIMR <25 had an increased post-RA caIMR (>25). To our knowledge, this is the first report on the evaluation of microcirculation function indicated by pre-RA caIMR in patients undergoing RA. It has been revealed that the RA debris containing atheromatous particles and platelet-rich tissue might be apt to induce embolic formation, subsequently resulting in clogging within the distal coronary microcirculation [29, 30]. We presumed that the resultant elevation of post-RA caIMR was mainly attributable to the microvascular embolization of atherosclerotic debris and associated thrombi [5]. Further, the possibility of microvascular spasm induced by RA cannot be excluded, even though nitroglycerin was continuously administered during the procedures.

In our study, we observed that among patients without microcirculation dysfunction reflected by pre-RA caIMR <25, the incidence of myocardial injury was significantly higher in those with post-RA caIMR ≥25 than in those with post-RA caIMR <25, while the rates of MI were comparable. This implies increased microvascular resistance resulting from micro-embolization of the debris generated during debulking of the lesion might cause detrimental effects such as myocardial injury. Most particles created by the RA procedure are <10 μm and have a mean diameter of 5 μm. They are smaller than normal, mature erythrocytes and can traverse coronary microvasculature cleared by the reticuloendothelial system [31]. However, in certain circumstances, the microdebris might be either too large to penetrate through the distal microcirculation or too abundant to be readily absorbed, and is consequently followed by increased caIMR, which indicates distal microvascular dysfunction [32]. However, procedure-related MI alone cannot adequately explain the difference observed between those with and without increased post-RA caIMR in our study, and more factors might be involved during the RA procedure. For instance, the release of adenosine from the ischemic myocardium due to the aggregation of the ablated microdebris could have made the RA procedure even more complicated.

Slow flow or no flow is not a rare phenomenon during RA, with reported rates varying from 7–10% [32, 33]. The incidence of instant TIMI flow grade <3 in the present study was 8.4%, and no flow occurred only in one case. Slow flow may lead to hemodynamic instability due to serious hypoperfusion, which is thought to be related to reduced coronary artery conductance [33, 34], which in turn is associated with increased occurrence of MI, rather than myocardial injury, as shown in our study. In our study, a reduction in coronary conductance was indicated by the finding that more patients with TIMI flow grade <3 had higher post-RA caIMR. RA did not definitely have a detrimental effect on the microcirculatory status in patients with post-RA TIMI flow grade 3, however, the observation that more patients with post-RA TIMI flow grade <3 had higher pre-RA caIMR indicates that the embolization of ablated microdebris and associated microthrombi subsequently followed by slow flow more likely occurred in patients with baseline coronary microcirculatory dysfunction.

In our adjusted analysis for various related variables, patients with maximum RA time of each pass had a significantly increased risk of the primary outcome. Prolonged rotational duration does not necessarily confer beneficial effects, which is consistent with a prior report which found that adopting an aggressive RA strategy did not offer advantage, and was sometimes even detrimental [35]. Notably, our study revealed that pre-RA caIMR ≥25 in the treated vessels, but not post-RA caIMR ≥25, was identified to be an independent predictor of the primary outcome with approximately a >3-fold increase in risk of the primary outcome among patients undergoing RA. The underlying coronary microcirculatory dysfunction indicated by an elevation of pre-RA caIMR was associated with a benign outcome in patients who underwent RA, and increased post-RA caIMR might only suggested to be a resultant slow flow.

## 5. Limitations

This study has several limitations. First, this was a single-center retrospective observational study, and the lack of a control group weakened the strength of the study’s implications. However, we performed a self-control analysis and measured caIMR in the reference vessels. Second, the decision to perform RA was at the discretion of the interventionist, and there was sustained improvement in the RA techniques across the cases. Thus, the results of our study should be interpreted with caution. Third, as the caIMR measurement was related to ${\mathrm{Pd}}_{\text{hyp}}$ and $V_{\text{diastole}}$, as mentioned above in the methods section, severe stenosis in the target vessels might influence the pressure and velocity in the distal vessel to some degree. Nevertheless, it has been reported that minimal microvascular resistance does not change with epicardial stenosis severity, and IMR is a specific index of microvascular resistance when coronary wedge pressure was taken into account [10, 36]. Despite the value of caIMR in our study was a corrected IMR following Yong formula, whether wire-derived IMR could be translated to caIMR deserve further study. Fourth, in this study, we only observed the instant and short-term impact of RA on coronary microcirculation, and long-term angiography follow-up data were not obtained. Analyzing the peri-procedural and in-hospital outcomes combined with long-term outcomes of RA affecting coronary microcirculation should be considered in future studies.

## 6. Conclusions

There were significant changes in the coronary microcirculation function of the target vessel after receiving RA, as indicated by a increase in post-RA caIMR compared with pre-RA caIMR. Post-RA TIMI flow grade <3 was more likely to be observed in patients with baseline coronary microcirculatory dysfunction. Patients receiving longer RA time of each pass and with pre-RA caIMR ≥25 had worse outcomes. In the future, further investigation of the impact of RA on long-term coronary microcirculatory function is warranted. To justify the clinical performance of caIMR, prospective and mode in-depth analysis should be designed also for short-term outcomes.

## Abbreviations

RA, rotational atherectomy; caIMR, coronary angiography-derived index of microvascular resistance; PCI, percutaneous coronary intervention; CFR, coronary flow reserve; CMR, coronary microvascular resistance; IMR, index of microvascular resistance; ACS, acute coronary syndrome; PPAE, peri-procedural adverse event; MI, myocardial infarction; rpm, revolutions per minute; TIMI, thrombolysis in myocardial infarction; cTn, cardiac troponin; CFD, computational fluid dynamics; MAP, mean aortic pressure.
